# A case of blurred vision due to coarctation of the aorta, an early clinical presentation, or just a coincidence?

**DOI:** 10.1097/MD.0000000000044081

**Published:** 2025-08-22

**Authors:** Zanhong Xiao, Jia Fu, Changjian Li, Jie Gong, Yong Zhang

**Affiliations:** a School of Medicine, Wuhan University of Science and Technology, Wuhan, China; b Department of Cardiology, Wuhan Children’s Hospital (Wuhan Maternal and Child Healthcare Hospital), Tongji Medical College, Huazhong University of Science and Technology, Wuhan, China.

**Keywords:** case report, child, coarctation of the aorta, hypertensive retinopathy, surgery, vision disorders

## Abstract

**Rationale:**

Coarctation of the aorta (CoA) is frequently misdiagnosed as primary hypertension. This is the first reported pediatric case of CoA presenting with both hypertension and blurred vision, highlighting an underrecognized diagnostic red flag.

**Patient concerns:**

An 8-year-old boy had blurred vision for 2 years and complained of “myopia,” admitted to the Department of Cardiology for “elevated blood pressure on physical examination for 2 days.”

**Diagnoses:**

Computed tomography angiography examination suggested CoA, which was confirmed by surgical and pathologic examination.

**Interventions:**

The cardiothoracic surgeon performed aortic arch angioplasty, aortic aneurysm resection, and anastomosis.

**Outcomes:**

Postoperatively, the child underwent pharmacologic antihypertensive and anticoagulant therapy. Six months later, his blood pressure was normal, and all medications were discontinued.

**Lessons:**

Blurred vision in children may signal hypertensive retinopathy from secondary causes like CoA. Early cardiovascular evaluation is critical to prevent irreversible end-organ damage.

## 1. Introduction

The coarctation of the aorta (CoA) is a congenital narrowing of the descending aortic arch, usually occurring in the distal left subclavian artery around the point of arterial catheter insertion,^[1]^ and is catheter-dependent congenital heart disease, accounting for approximately 4% to 6% of congenital heart disease.^[2–4]^ The main pathophysiologic changes were increased left ventricular afterload, increased blood pressure (BP) proximal to the arterial stenosis, and decreased distal BP. Therefore, for the upper and lower limbs, a systolic pressure step difference ≥ 20 mm Hg can make a clinical diagnosis and further confirm that the diagnosis needs to be combined with computed tomography (CT), magnetic resonance, or aortography, and other imaging findings.^[5]^ After the diagnosis of CoA was precise, the primary goal of its treatment was to release the stenosis, reestablish normal blood flow channels in the aorta, restore normal BP and circulatory function, and minimize the pressure step difference around the narrowing and complications. Current treatment options for CoA include surgical and interventional treatments.^[6]^ In 1944, CoA 1st successful surgical treatment was reported, making surgery the primary method for CoA.^[7]^ The surgical technique has been upgraded from the classic end-to-end anastomosis of stenotic segments to patch enlargement angioplasty. The incidence of surgical complications is gradually decreasing, with most children with CoA recovering after surgery.^[8]^

Although there were well-established protocols and a good prognosis in the treatment of CoA, the difficulty was early diagnosis and recognition. The simple type of CoA was usually only detected briefly because it was generally asymptomatic in the early stages. At the same time, there might be an early indication through the symptoms of hypertensive target organ damage. The eye is a unique sensory organ because it allows direct observation of the retinal microcirculation and, therefore, provides a window to detect microvascular changes associated with the development of various cardiovascular diseases, such as arterial hypertension or coronary artery disease.^[9]^ Once, a scholar reported a case of corkscrewing of retinal arterioles leading to the diagnosis of CoA in a 16-year-old male patient.^[10]^ However, there were a few reports of CoA patients with vision disorders or blurred vision as the 1st symptom. Bilitardo et al previously reported a posterior reversible encephalopathy syndrome caused by CoA in a child with visual changes but with other central nervous system manifestations.^[11]^ Here, we wrote a case of a child with blurred vision who was finally diagnosed with CoA. Surgical treatment was also performed, and good results were achieved. Due to the small number of cases, we could not determine whether the association between blurred vision and CoA was random. Still, this case was suggestive and informative for the diagnosis of CoA.

## 2. Case report

**Figure 1.**
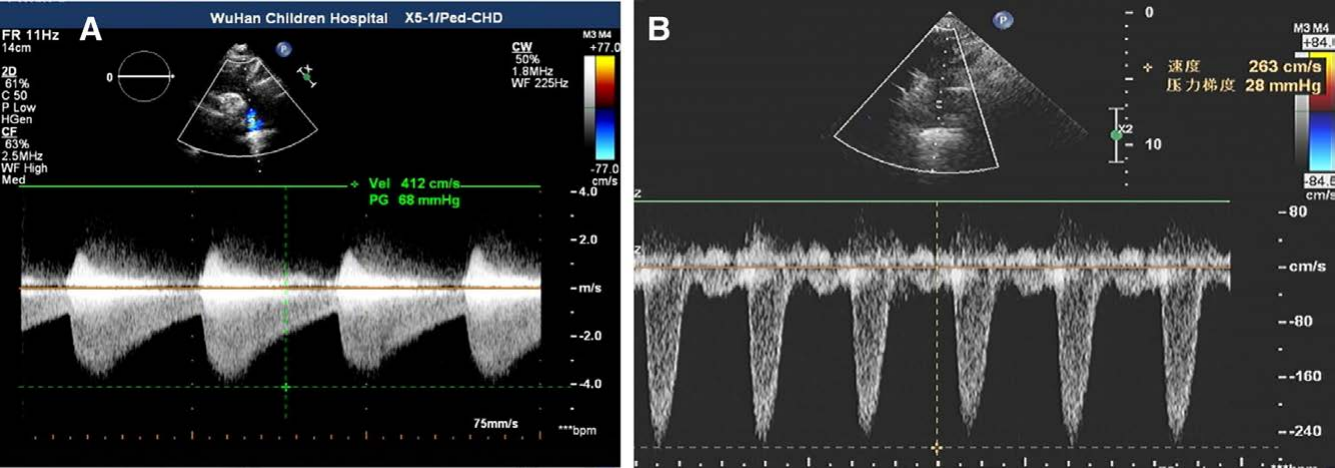
Pulsed Doppler assessment of blood flow velocity in CoA. (A) Pulse Doppler ultrasound at the site of CoA before surgery showed an acceleration of flow up to 4.12 m/s. (B) Postoperative pulsed Doppler ultrasound of the CoA site showed a decrease in blood flow velocity to 2.63 m/s. The 2D cardiac ultrasound data were as follows: EDV 71 mL, ESV 22 mL, SV 49 mL, EF (%) 69%, LVIDd 40.32 mm, LVIDs 24.76 mm, LVPW 7.5 mm, and IVS 10 mm. The morphology and structure of the heart valves were normal, and no significant regurgitation was detected. CoA = coarctation of the aorta, EDV = end-diastolic volume, EF = ejection fraction, ESV = end-systolic volume, IVS = interventricular septum, LVIDd = left ventricular internal diameter diastolic, LVIDs = left ventricular inner diameter systolic, LVPW = left ventricular posterior wall, SV = systolic volume.

**Figure 2.**
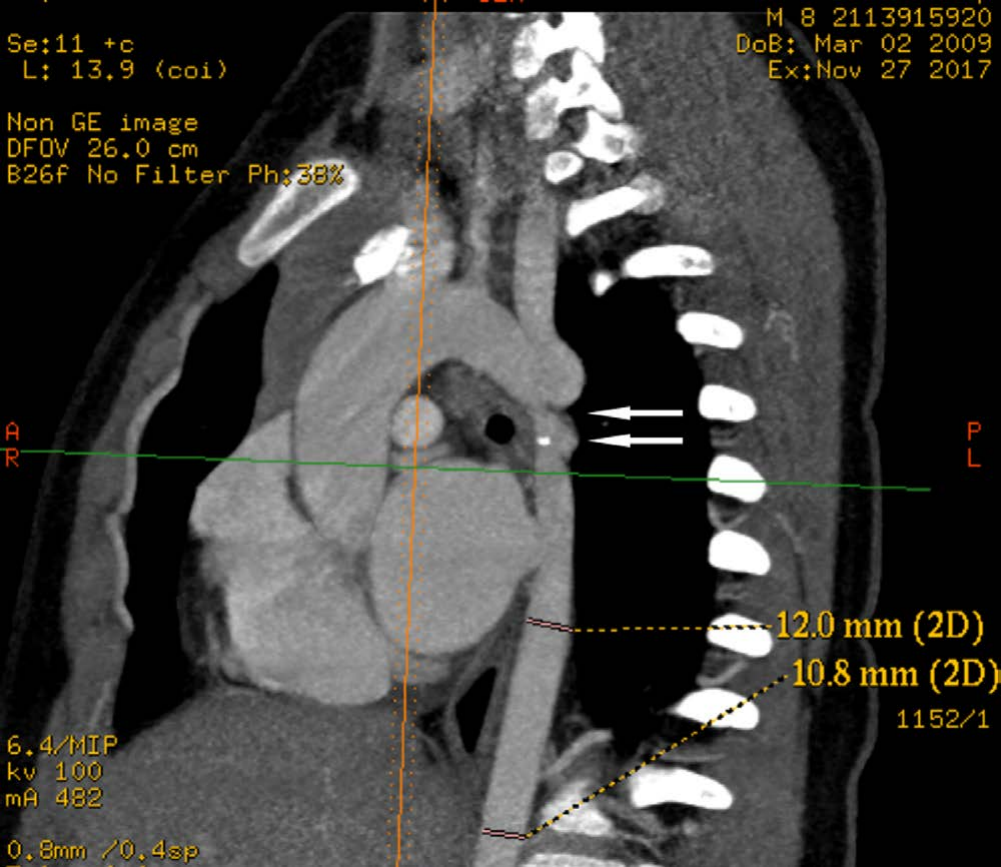
Cardiac CT angiography. The CT angiography suggested CoA (the upper arrow) with aneurysmal dilatation (the lower arrow). Two stenoses of the descending aorta can be seen, with a width of 3 to 4 mm at the narrowest point. CoA = coarctation of the aorta, CT = computed tomography.

An 8-year-old boy had blurred vision for 2 years and complained of “myopia,” but his family did not take it seriously or manage it. He was admitted to the Department of Cardiology for “elevated BP on physical examination for two days.” It was repeatedly confirmed that the child presented only with blurred vision that lasted for 2 years, and he never experienced dizziness, headache, chest pain, eye pain, or fatigue. Physical examination revealed body mass index = 42.3 kg/m^2^, upper limbs BP was 188/138 mm Hg, lower limbs BP was 90/54 mm Hg, and no significant heart murmur was heard in the chest and back. The child was given oral and intravenous antihypertensive medication, but the antihypertensive effect was unsatisfactory. Blood routine examination showed a white blood cell count of 6.77 × 10^9^/L, a neutrophil ratio of 47.4%, a lymphocyte ratio of 41.7%, a red blood cell count of 5.11 × 10^12^/L, a hemoglobin of 135 g/L, and a platelet count of 446 × 10^9^/L. Combined T cell-B cell-NK cell test showed CD4 + T-cell% was 39.91%, Th/Ts: 2.23, and the rest were in the normal range. Erythrocyte sedimentation rate, lactate, immunoglobulin level, cortisol (8 am), cortisol (8 pm), C-reactive protein, thyroid function, blood ammonia, liver and kidney function, electrolytes, cardiac enzymes, lipids, blood glucose, coagulation function, adrenocorticotropic hormone, a complete set of sex hormone tests, as well as routine urine and fecal tests were all within normal limits. The catecholamine qualitative test was negative. T-cell spot was negative. Cardiac ultrasound suggests congenital heart disease: CoA, accelerated antegrade flow in the descending aorta with a peak flow velocity of 4.1 m/s and differential pressure of 68 mm Hg; no significant structural cardiac abnormalities were observed (Fig. 1A). The CT angiography suggested CoA (Fig. 2). An ophthalmologist was consulted, and myopia was ruled out. Completed fundus photography showed a well-defined optic papilla with average luster, an arteriovenous ratio of 1:2, enhanced vessel wall reflexes, and no retinal hemorrhage or exudate; a diagnosis of stage I hypertensive retinopathy was made. Therefore, a diagnosis of CoA, secondary hypertension, and hypertensive retinopathy was made, and the patient was referred to cardiothoracic surgery for continued treatment.

**Figure 3.**
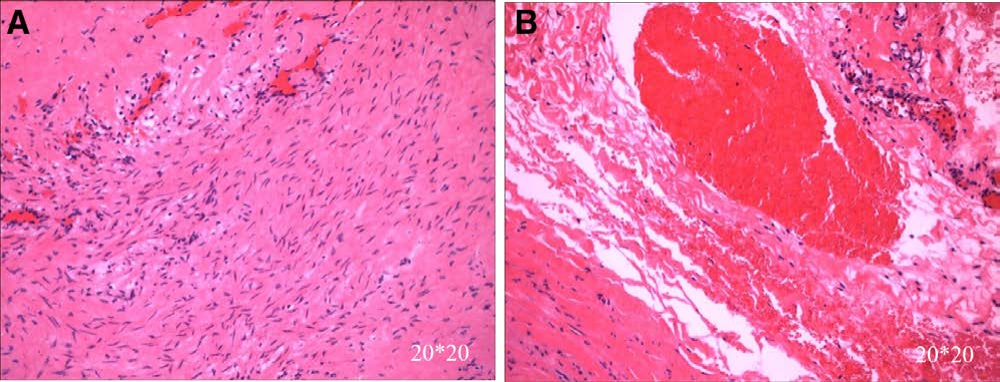
Microscopic findings and pathological diagnosis of the submitted specimen. Pathological examination revealed limited stenosis within the aortic vessel wall, elastic fibrous degeneration of the arterial wall, and intimal thickening. (A) Arterial wall elastic fibrous degeneration and (B) vasodilatation and hemorrhage in localized areas.

**Figure 4.**
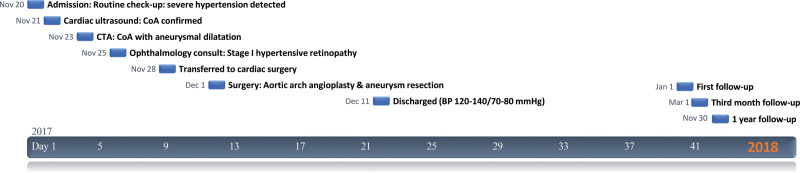
Timeline of patient admission to follow-up. BP = blood pressure, CoA = coarctation of the aorta, CT = computed tomography.

After the transfer, the Department of Cardiothoracic Surgery performed aortic arch angioplasty, aortic aneurysm resection, and anastomosis. Intraoperatively, the descending part of the aortic arch was exposed, and an aneurysm was seen dilated at the beginning of the descending aorta behind the distal arterial ligament of the aortic arch, about 2.0 × 2.0 × 0.5 cm, with a thin aneurysm wall and a narrower aortic arch and descending aorta wall than in an average child. Longitudinal incision of the anterior wall of the aortic aneurysm revealed a fragile anterior wall and a thickened posterior wall with thickening of the aortic wall at the superior and inferior ends of the aortic aneurysm, creating a stenotic thickness of approximately 4 mm internal diameter. The anterior wall of the aortic aneurysm was excised, and a 3.5 × 4 cm surgical bio patch (Bairen Medical Technology, Beijing, China) was taken to reconstruct the descending aortic arch through an extended incision above and below the stenosis. Histopathology has shown that some descending aortic aneurysm tissue exhibits partial tissue degeneration with localized areas of vasodilation and hemorrhage (Fig. 3). Postoperative diagnosis of CoA and the aortic aneurysm was made, and the cardiac ultrasound was repeated: aortic arch descending pressure difference of 26 mm Hg (Fig. 1B); the rest was not unique. On the 1st day after surgery, the child’s BP was 150–160/90–100 mm Hg in the upper limbs and 120/90 mm Hg in the lower limbs. One week after surgery, the BP fluctuated at 140–150/100–110 mm Hg, and nitroprusside pumping was discontinued. Two weeks after surgery, the BP fluctuated at 120–140/70–80 mm Hg, and discharge was arranged. After release, he continued oral antihypertensive therapy with amlodipine besylate (5 mg/daily), spironolactone (1–2 mg/kg/daily), and hydrochlorothiazide (1–2 mg/kg/daily), and anticoagulation with aspirin (3–5 mg/kg/daily). After 3 months, the BP was in the normal range (110–120/70–80 mm Hg). All medication was discontinued, followed by regular follow-up visits with outpatient and self-test BP in the normal range, and the child did not complain of blurred vision again (Fig. 4).

## 3. Discussion

According to the clinical utility, CoA is mainly advocated as a simple and complex type, with the simple type accounting for more than 90% of the stenotic segments located in the aortic isthmus without combined arteriovenous and other intracardiac malformations.^[12,13]^ Here, we reported managing an 8-year-old child with a simple type of CoA. The difference in BP between the upper and lower extremities in children readily suggested the possibility of CoA, which, together with diagnostic imaging, ultimately confirmed the diagnosis of CoA. After the precise diagnosis, the child underwent immediate surgery and achieved good results. Transcatheter stenting has become the intervention of choice for some children with CoA weighing ≥ 25 kg.^[14]^ However, the surgical options for CoA remain controversial, with the need for re-intervention to expand the stent after intervention as the child grows.^[15,16]^ Hence, the risks of repeated transcatheter interventions need to be weighed against more invasive surgical procedures. In clinical practice, stenting is generally recommended for children who can accept stents scaled up to adult size.^[14]^ In this case, the child was 8 and had a combined aneurysm, which we treated surgically.

With the improvement of medical treatment, significant progress has been made in diagnosing and treating CoA. However, due to the insidious nature of the onset of the disease, early recognition of CoA has become increasingly important. If diagnosed early, complications can be prevented, and medical expenditures can be reduced.^[17]^ The child had developed blurred vision 2 years earlier, and the possibility of CoA would have been detected earlier if sufficient attention had been paid. In our case, we hypothesized that blurred vision in the child was one of the symptoms of hypertensive retinopathy due to the following reasons: 1st, elevated aortic pressure (up to 188/138 mm Hg) leads to vasoconstriction of the retinal arterioles and hypertrophy of their walls (funduscopic examination shows an arteriovenous ratio of 1:2 and enhanced light reflex). Prolonged hypoperfusion disrupts retinal autoregulation and induces microvascular ischemia, which is subjectively manifested as impaired visual acuity.^[18]^ Second, CoA correction confirms that BP and vision problems are resolved. Finally, ophthalmologist consultation and systematic ophthalmologic examination evaluation ruled out other possible causes of blurred vision: refractive examination ruled out significant refractive error, optical coherence tomography showed no macular edema; cranial magnetic resonance imaging showed no encephalopathy syndromes, optic neuropathy, or demyelinating lesions; and metabolic testing showed normal lipids, blood glucose, hormone level testing, and thyroid function, ruling out diabetes and endocrine disorders. This evidence suggests that hypertensive retinopathy was the most likely cause of the visual impairment in this case. It has been reported that a child with COA was detected and eventually diagnosed by routine fundoscopy, which is consistent with our report.^[19]^ However, it is essential to emphasize that vision changes due to COA are not necessarily a direct result of fundus lesions due to hypertension. In another paper, a child with COA-induced changes in visual acuity had a normal fundoscopic examination, which was ultimately determined to be the result of central nervous system damage due to posterior reversible encephalopathy syndrome.^[11]^ In children with vision problems, neurologic causes must be ruled out. Hence, it is essential to emphasize that the lack of evidence on whether blurred vision and CoA were necessarily linked in this case is a limitation. Therefore, we shared this compelling case to provide more inspiration and hints for diagnosing and treating CoA rather than confirming the disease by specific symptoms.

The 1st inspiration was that the onset of hypertension was insidious. Due to the timing and conditions of outpatient visits, it was often impossible to think of or measure the BP in the patient’s extremities, resulting in misdiagnoses. In this case, the etiology of hypertension was further defined after hospitalization. Arterial BP measurements of the extremities showed significant differences between upper and lower limbs, followed by cardiac ultrasound and aortic CT angiography to confirm the diagnosis. Therefore, we recommend that comparative measurement of BP in the extremities of children and adolescents with hypertension be given high priority to rule out these conditions, especially in children with abnormally high BP. Second, in the early stages of hypertension, the symptoms were not obvious, and, unlike adults, most children did not have their BP monitored regularly. Therefore, pediatricians cannot ignore some hypertensive target organ damage symptoms. In this case, the child had a history of blurred vision. Still, both the child and the family thought it was due to “myopia.” They did not systematically examine and treat it, thus failing to detect the early detection of hypertension and, therefore, to evaluate the cause of hypertension. Hence, hypertension should be considered in children with blurred vision. Although the number of cases reported in this case was small and there was randomness in the association between blurred vision and CoA in the case, this did not prevent the pediatrician from assessing the child and not ignoring any symptoms, even if they were not the child’s chief complaint or distress. Of course, in this case of hypertension, some routine tests were performed for differential diagnosis, as it was necessary to exclude secondary causes of hypertension in children. We excluded secondary hypertensive disorders related to the kidney, endocrine, or taking particular medications, and finally diagnosed congenital CoA.

## 4. Conclusion

Overall, with advances in surgery, the surgical treatment of CoA tends to have a better prognosis. The current difficulty in treating the disease lies in early diagnosis and recognition, a challenge for pediatricians. Comparative measurement of extremity BP is the most basic and vital method to detect CoA in children and adolescents with hypertension. However, in children without symptoms of hypertension, our pediatricians often need to see and focus on signs of hypertensive target organ damage that are easily overlooked.

## Author contributions

**Data curation:** Zanhong Xiao.

**Methodology:** Jie Gong.

**Visualization:** Changjian Li.

**Writing – original draft:** Jia Fu, Changjian Li.

**Writing – review & editing:** Yong Zhang.
